# Same but Different? Comparing the Epidemiology, Treatments and Outcomes of COVID-19 and Non-COVID-19 ARDS Cases in Germany Using a Sample of Claims Data from 2021 and 2019

**DOI:** 10.3390/v15061324

**Published:** 2023-06-05

**Authors:** Eva Bernauer, Felix Alebrand, Manuel Heurich

**Affiliations:** 1 BinDoc GmbH, Karlstraße 3, 72072 Tübingen, Germanymanuel.heurich@bindoc.de (M.H.); 2 Clinic for Cardiology and Pneumology, Georg-August-Universität Göttingen, 37075 Göttingen, Germany

**Keywords:** ARDS, COVID-19, comorbidities, treatments, adverse events, mortality

## Abstract

Background: Acute respiratory distress syndrome (ARDS) is a severe lung condition that can be caused by a variety of underlying illnesses. Due to SARS-CoV-2, the number of cases with ARDS has increased worldwide, making it essential to compare this form of acute respiratory failure with classical causes of ARDS. While there have been several studies investigating the differences between COVID-19 and non-COVID-19 ARDS in early stages of the pandemic, little is known about the differences in later phases, especially in Germany. Aim: The aim of this study is to characterize and compare the comorbidities, treatments, adverse events, and outcomes of COVID-19-associated ARDS and non-COVID-19 ARDS using a representative sample of German health claims data from the years 2019 and 2021. Methods: We compare percentages and median values of the quantities of interest from the COVID-19 and non-COVID-19 ARDS group, with p-values calculated after conducting Pearson’s chi-squared test or the Wilcoxon rank sum test. We also run logistic regressions to access the effect of comorbidities on mortality for COVID-19 ARDS and non-COVID-19 ARDS. Results: Despite many similarities, we find that that there are some remarkable differences between COVID-19 and non-COVID-19 ARDS cases in Germany. Most importantly, COVID-19 ARDS cases display fewer comorbidities and adverse events, and are more often treated with non-invasive ventilation and nasal high-flow therapy. Conclusions: This study highlights the importance of comprehending the contrasting epidemiological features and clinical outcomes of COVID-19 and non-COVID-19 ARDS. This understanding can aid in clinical decision making and guide future research initiatives aimed at enhancing the management of patients afflicted with this severe condition.

## 1. Introduction

Acute respiratory distress syndrome (ARDS) is a severe and potentially life-threatening lung condition that is characterized by widespread inflammation, lung injury, and pulmonary edema, resulting in difficulty in breathing, decreased oxygen levels, and potentially, hypoxemia-related damage throughout the body. ARDS typically develops as a complication of a wide spectrum of underlying injury or illnesses, such as pneumonia, sepsis, trauma, and aspiration [1]. Even though ARDS diagnosis and therapy has improved significantly over the last few decades, its associated morbidity and mortality remain high [2].

Since the beginning of the SARS-CoV-2/COVID-19 (COVID-19) pandemic, the incidence of ARDS cases has increased drastically, as ARDS is an important complication of COVID-19. An early global literature survey reported that around 33% of hospitalized patients with COVID-19 developed ARDS in 2020 [3]. Given the significant impact of COVID-19 on ARDS, many authors have posed the question of whether COVID-19-induced ARDS differs from classical forms of non-COVID-19 ARDS [4]. While many clinical parameters of COVID-19-associated ARDS and classical ARDS seem similar, and the current guidelines for the management of COVID-19 ARDS are mostly in accordance with pre-existing evidence-based ARDS strategies [5,6,7], some authors have emphasized that COVID-19 ARDS may have some different pathophysiological features, comorbidities, and outcomes, as well as a different onset of symptoms and clinical course, compared to non-COVID-19 ARDS [4,8]. Its onset of symptoms seems to be prolonged at 8–12 days compared to 7 days in classical ARDS [4]. Besides the classical symptoms of ARDS such as fever, cyanosis, and respiratory distress, which cannot be adjusted by normal oxygen therapy, it often shows an atypical lack of dyspnea, which might be explained by the neurotoxic characteristics of the virus [9]. Such as in classical causes of ARDS, age seems to be a major risk factor for developing ARDS with COVID-19. Two small cohort studies suggested that COVID-19 ARDS patients might be older and have a higher prevalence of diabetes and obesity than patients with classical ARDS [10,11]. Others found diverging results [12,13]. Looking at the treatment, several studies suggested that high-flow nasal cannula therapy is more suitable for patients with COVID-19 ARDS than for those with classical ARDS [4]. In 2020, patients with COVID-19 ARDS also experienced prolonged ICU stays and mechanical ventilation compared to those with non-COVID-19 ARDS [11].

In Germany, there is only limited evidence on the epidemiology of ARDS during and prior to the COVID-19 pandemic. Although three studies investigated the comorbidities and characteristics of COVID-19 ARDS patients in Germany in 2020, they did not compare them to non-COVID-19 ARDS cases in the same time span [14,15,16]. Furthermore, at the time of writing this paper, there are no data on the characteristics, comorbidities, and treatments of COVID-19 ARDS in later phases of the pandemic after 2020 in Germany. This is especially interesting since vaccination started in Germany in December 2020, and at the beginning of 2021, new virus mutants such as the (B.1.1.7) alpha variant and, over the course of the year, primarily B.1.612.2 (delta) became more prominent in Germany [17,18,19].

In this paper, we aim to use a representative sample of anonymized German health claims data to assess and compare the comorbidities, treatments, adverse events, and outcomes of COVID-19 ARDS patients in 2021 to those of non-COVID-19 ARDS patients in 2021 and 2019. By employing this comprehensive approach, our objective is to advance our understanding of the epidemiology of ARDS in Germany while elucidating the potential distinctions and similarities between COVID-19-induced and non-COVID-19-induced ARDS, considering the previous partly conflicting findings in the literature. The findings of this study have the potential to make contributions to the future development of novel strategies for managing patients affected by these conditions.

## 2. Materials and Methods

### 2.1. Source of Data

The primary data source was a sample of anonymized routine claims data from Germany, covering about 10 percent of the German in-patient population. Since the data were anonymized, informed consent and ethical approval were not required. The sample was non-random, and the representativeness of the data must be assessed (cp. Section 2.4).

### 2.2. Codes Regarding Patient Characteristics, Diagnoses, Procedures, and Outcomes

The patient-level claims data contained: information on gender and age (here, divided into 21 age groups for anonymization purposes) of the patient; the main diagnosis and all secondary diagnoses according to the International Classification of Diseases (ICD-10); procedures according to the German Procedure Classification (OPS Codes, see BfArM); the patient’s length of stay in hospital (LOS) and in an intensive care unit (LOS ICU); as well as the patient’s admission and dismissal reasons, including whether the patient died or whether he/she was transferred to another hospital. Thus, it is possible to extract all relevant information for the present analyses.

### 2.3. Parameters, Definitions, and Study Outcomes

We defined the sample of ARDS cases using the criterion of ICD-10-Code J80.x, being the main or the secondary diagnoses. J80.x differentiates severities of ARDS as defined by the Berlin definition [20], which foresees three plus one categories based on the extent of hypoxemia: mild (J80.1, PaO_2_/FiO_2_ (the ratio of the partial pressure oxygen in blood (PaO_2_), and the fraction of oxygen in the inhaled air (FiO_2_) is known as the Horovitz quotient and is used to assess the lung function in patients) = 201–300 mmHg, and PEEP ≥ 5 cmH_2_O), moderate (J80.2, PaO_2_/FiO_2_ = 101–200 mmHg, and PEEP ≥ 5 cmH_2_O), severe (J80.3, PaO_2_/FiO_2_ = ≤ 100 mmHg, and PEEP ≥ 5 cmH_2_O), and indeterminate (J80.9). COVID-19 ARDS cases were identified with ICD-10-Code U07.1, U07.2, and B97.2. ICD-10 B97.2 was used as a COVID-19 code at the beginning of the pandemic and occasionally thereafter, before U07.x was introduced. We added it here to the COVID-19 category to prevent COVID-19 cases from being included in the non-COVID-19 category, but the number of these cases was small (3.2% of the COVID-19 cases in 2021) and did not change the findings. The “year” was the year of the dismissal of a patient. The present study primarily focused on the year 2021 for the comparison of COVID-19 and non-COVID-19 ARDS, as in 2020 there was still a mixed coding of COVID-19 cases. The cases from 2019, as a pre-COVID-19 year, were relevant to establish the robustness of the non-COVID-19 results.

Adipositas was defined by ICD-10-Code E66, while comorbidities were defined via the components of the Charlson Index [21], and the Charlson Score was calculated with the updated weighting algorithm by Quan [22]. As risk factors for ARDS, we considered pneumonia (ICD-10: J09-J18, A48.1, J85, J86, U69.0), sepsis (ICD-10: A40, A41, A42.7, B37.7, A26.7, A28.2, A54.8, B00.7, A24.1, A39.2-.4, A22.7, A20.7, A21.7, O75.3, A48.3, R65), trauma (ICD-10: S; T00: T07, T14), aspiration (W78, J69.0, J69.1, J69.8, J95.4), cancer (ICD-10: C), thoracic surgery (ICD-10: J95.1; OPS: 5-32, 5-33, 5-34), and acute pancreatitis (ICD-10: K85, K86.0, K86.1, K87.1). For the ventilation procedures, we assumed a ranking of: no ventilation, non-invasive ventilation (via mask or helmet, OPS code 8-706), nasal high-flow therapy (NHF, OPS 8-713), invasive ventilation via intubation (OPS code 8-701, 8-704), and tracheotomy (OPS 5-311, 5-312). We coded whether these ventilation procedures were applied at all, and whether they were the maximum level given. We also reported the number of cases with no OPS ventilation code, when ventilation was started in a prior hospital or an ambulance. Other than ventilation, we considered ECMO (OPS code 8-852.0/3/5/6) as a treatment; additionally, we coded dummies on dialysis (OPS 8-853, 8-854, 8-855), blood transfusions (8-800), cardioversion (8-640.0), defibrillation (8-640.1), and reanimation (8-771, 8-779). As adverse events, we considered and defined septic shock (ICD-10 R57.2), cardiogenic shock (R57.0), hypovolemic shock (R57.1), acute kidney failure (stad. 2/3; N17.02/.03, N17.12/13, N17.22/23, N17.82/83, N17.92/93), myocardial infarction (I21, I22), cardiac arrest (I46, R00.3), acute liver failure (K72.0), acute ulcer or gastrointestinal bleeding (K92.2, K25.0, K25.1, K25.2, K25.3, K26.0, K26.1, K26.2, K26.3, K29.0), DIC (D65.1), pulmonary embolism (I26), pneumothorax and lung collapse (J93, J98.1), pleural effusion (J90, J91), and delirium (F05). Further outcomes included length of stay in hospital (LOS) or in an intensive care unit (LOS ICU); in-hospital mortality or death (code 79); and transfer to another hospital (code 6x/8x, according to §21 KHEntG) as a dismissal reason.

### 2.4. Research Process and Representativity

During the data collection period (29 June 2022), our dataset comprised 2.54 million patients from 165 hospitals for the year 2019, and 1.6 million patients from 116 hospitals for the year 2021, all obtained from the BinDoc database. To ensure consistency in our sample of hospitals between the two years, the number of hospitals was reduced to 112. Consequently, the total number of patients for 2019 and 2021 was 1.8 million and 1.6 million, respectively, representing approximately 10 percent of the German in-patient population. From this pool, we specifically focused on patients diagnosed with ARDS as per our defined criteria, resulting in a reduced sample of 1507 cases in 2019 and 4046 cases in 2021. However, due to missing data in the secondary codes used to identify COVID-19 cases, the actual number decreased further to 1486 ARDS cases in 2019 and 3928 ARDS cases in 2021. Among the latter, 2654 cases were attributed to COVID-19, while the remaining 1274 cases were not COVID-19 related.

As this study was based on a non-random sample of German routine claims data, it was crucial to establish representativity. Therefore, we compared key summary measures of our data on COVID-19 ARDS cases from 2020 with key summary measures from Sagoschen et al. (2022) [16], who looked at the entire population of COVID-19 ARDS cases from German claims data in 2020. In Table 1, we compare the median age group in our data with the median age in Sagoschen et al., as well as the percentage of female patients, the percentage of obese patients, the percentage of patients with pneumonia, the percentage of patients with mechanical ventilation, the percentage of patients who received ECMO treatment, LOS, and mortality. We found that our 10 percent sample is quite representative, as the sample’s median values or percentages are close to the true values in the population according to Sagoschen et al. [16].

### 2.5. Statistical Methods

**Figure 1:**
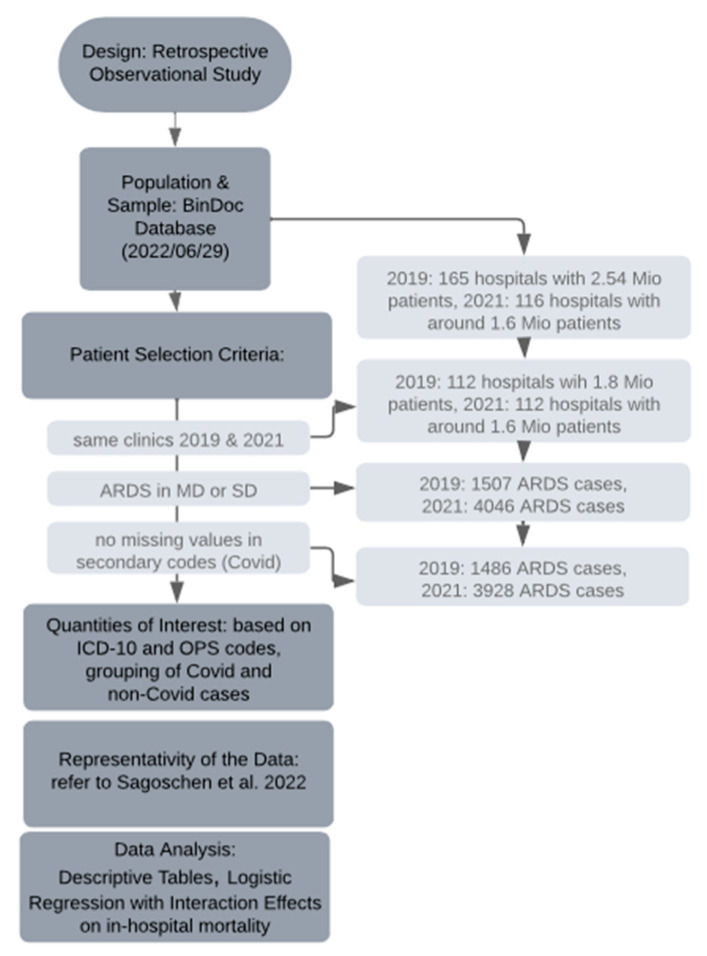
Timeline of the research process; for Sagoschen et al., 2022, see [16].

We considered three groups of patients: ARDS patients with a diagnosis of COVID-19 in 2021; ARDS patients with no diagnosis of COVID-19 in 2021; and ARDS patients with no COVID-19 in 2019. We compared the COVID-19 ARDS patients from 2021 with the other two groups: for all the above measures (patient characteristics, treatments, outcomes, etc.), we reported percentages and median values as well as *p*-values after carrying out Pearson’s chi-squared test or the Wilcoxon rank sum test. We used R software version 4.2.2 with the packages comorbidities version 1.0.5 [23] and gtsummary version 1.6.2 [24] (see Figure 1 for the research process).

Using a logistic regression, we examined the relative importance of patients’ comorbidities for in-hospital mortality as an interaction within the COVID-19 and the non-COVID-19 group in 2021 (AIDS was left out due to extremely few positive observations). We considered comorbidities next to gender, age, and adipositas, removed all rows with missing values, and checked for influential observations and multicollinearity (there was none). If the interaction term was significant on the 5% level (*p*-value of < 0.05, two-sided), it indicated that the factors have a different influence among COVID-19 and non-COVID-19 cases [25]. The statistical significance of the interaction effect for each observation is plotted in the Supplementary Material [26]. We additionally present figures on predicted probabilities for (i) no comorbidities and for (ii) a “worst-case-scenario” in COVID-19 ARDS cases, including the strongest positive predictors. We used the R packages forestplot version 3.1.1 [27], DAMisc version 1.7.2. [28], and ggeffects version 1.1.4 [29].

Sensitivity analyses: as a robustness check, we used restricted cubic splines in the age variable; additionally, we ran models that further included all adverse events, treatments, etc., which was followed by the stepwise forward and backward deletion of covariates with the built in step function in R (see Supplementary Material).

## 3. Results

### 3.1. Baseline Characteristics

In 2021, there were 3928 ARDS patients in our database, of which 2654 were COVID-19 ARDS and 1274 were non-COVID-19 ARDS cases (Table 2). During this timeframe, the prevailing SARS-CoV-2 variants in Germany were B.1.1.7 (alpha), B.1.612.2 (delta), and, by the end of 2021 in December, increasingly B1.1.529 (omicron) [17,18,19]. Looking at the comparison of the baseline characteristics of COVID-19 ARDS and non-COVID-19 ARDS, there were no significant differences in age and obesity between the groups. The percentage of female patients in the COVID-19 ARDS group was slightly less than in the non-COVID-19 group. The admission causes for COVID-19 patients were mainly and significantly emergency admission causes as compared to non-COVID-19 ARDS patients. COVID-19 ARDS patients had fewer comorbidities, especially cardiovascular diseases, but there were more diabetes patients without complications in this group. Naturally, the set of risk factors was more diverse in non-COVID-19 ARDS than in pneumonia-driven COVID-19 ARDS, although, remarkably, even COVID-19 ARDS patients experienced sepsis, trauma, etc. (cp. Table 2).

As for the treatments and outcomes (Table 3), the COVID-19 group received non-invasive ventilation more often and had less dialysis, cardioversions, and blood transfusions, while non-COVID-19 patients received more invasive ventilation. There were no differences in the number of patients who underwent tracheostomy and ECMO treatment. Adverse events were coded more often in the non-COVID-19 group, such as shock, acute severe kidney failure, gastrointestinal bleeding, and respiratory complications. In 2021, COVID-19 ARDS had a significantly lower mortality rate (45.9% vs. 51.2%) than non-COVID-19 ARDS. However, the transfer rate to another hospital was significantly higher (25.55% vs. 18.13%).

Comparing COVID-19 ARDS cases in 2021 with non-COVID-19 ARDS cases prior to the pandemic in 2019 showed similar results in terms of comorbidities, treatments, and adverse events (last column in Table 2 and Table 3). The percentage of obese patients seems to have increased from 2019 to 2021 in the non-COVID-19 ARDS group. Divergent from the 2021 comparison, peptic ulcers and renal disease became significant in the 2019 comparison and occurred more often in 2019. ECMO treatment was also used more often in non-COVID-19 cases in 2019, which may be explained by capacity limitations in 2021. Interestingly, the risk of pulmonary embolism was significantly lower in non-COVID-19 ARDS in 2019.

### 3.2. Logistic Regression

**Figure 2:**
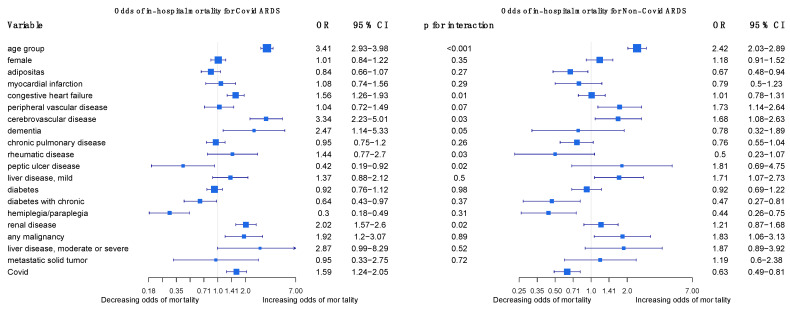
Odds of in-hospital mortality for COVID-19 ARDS and non-COVID-19 ARDS patients in Germany 2021.

**Figure 3:**
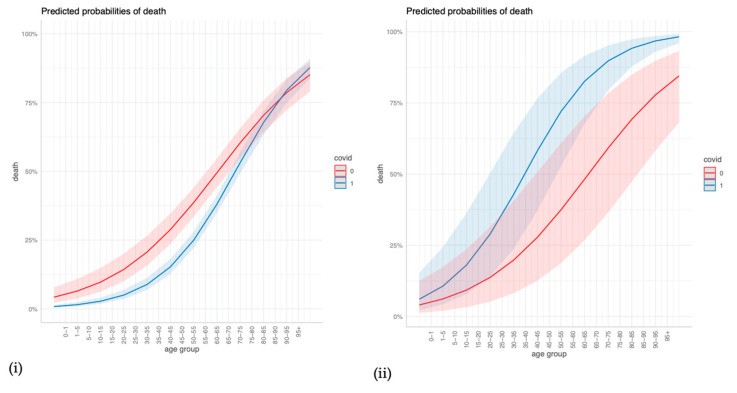
Predicted probabilities of death for different values of age when comparing COVID-19 and non-COVID-19 ARDS for (**i**) no comorbidities, and (**ii**) a COVID-19 ARDS “worst-case-scenario”.

The regression outcomes in Figure 2 show that a higher age, cerebrovascular disease, and any malignancy significantly increase the odds of dying in COVID-19 and non-COVID-19 ARDS cases alike, but age and cerebrovascular disease increase the odds even more so in COVID-19 cases. Congestive heart failure, dementia, and renal disease significantly increase the odds of dying in COVID-19 cases but are insignificant in non-COVID-19 cases. Diabetes with chronic complications and hemiplegia/paraplegia seem to reduce the odds of dying in both non-COVID-19 and in COVID-19 cases. The sensitivity analyses in the Supplementary Material confirm that age, dementia, renal disease, and any malignancy are strong and robust positive predictors of mortality in COVID-19 ARDS, and they confirm the differences in age, renal disease, and congestive heart failure between COVID-19 ARDS and non-COVID-19 ARDS (Figures S1–S3). For a more intuitive illustration, Figure 3 displays how the predicted probabilities change with age when comparing COVID-19 and non-COVID-19 ARDS in (i) the case of no comorbidities, and (ii) a “worst-case-scenario” for COVID-19 ARDS, with the dummy predictors of dementia, renal disease, and congestive heart failure set to one.

## 4. Discussion

Until now, there have been limited data available on the epidemiology of COVID-19 and non-COVID-19 ARDS in Germany. Our study provides a concise overview of the characteristics, comorbidities, and treatments of COVID-19 ARDS patients in Germany in 2021 and compares them to those of non-COVID-19 patients in 2021 and 2019.

### 4.1. Epidemiology and Comparison of Comorbidities in COVID-19 and Non-COVID-19 ARDS

In a previous observational study that investigated 106 COVID-19 ARDS cases in five major ICUs in Germany in 2020, 24.5% of patients had diabetes, 15.1% had chronic pulmonary disease, 14.2% had heart failure, 15% had chronic renal failure, and 11% had cancer [15]. Our data revealed a similar comorbidity pattern for COVID-19 ARDS patients in Germany in 2021, with a lower proportion of cancer (3.84%) and a slightly higher proportion of patients with congestive heart failure (21.97%).

Studies on the epidemiology and comorbidities of non-COVID-19 ARDS patients in Germany during and prior to the pandemic in 2021 and 2019 are rare. The LUNG SAFE study in 2016 was the largest study investigating the epidemiology of ARDS to date, including 3022 ARDS patients from 459 ICUs in 50 countries over a 4-week period in 2014. The study reported that 8.5% of patients had an active neoplasm, 21.7% had diabetes, 10.4% had chronic cardiac failure, and 10.1% had chronic renal failure [30]. In our study, we identified 1274 non-COVID-19 ARDS cases in 2021 and 1486 non-COVID-19 cases in 2019 in our sample of German claims data. Comparing non-COVID-19 ARDS cases in 2021 to the results of the LUNG SAFE study, we found a similar proportion of patients with cancer but a significantly higher proportion of patients with congestive heart failure, diabetes, and chronic renal failure. This trend was also observed in 2019, where the comorbidities were even more prevalent than in 2021. As there may be regional differences in the prevalence and diagnosis of certain diseases, this may suggest that non-COVID-19 ARDS patients in Germany have more comorbidities than previously thought.

Although earlier studies indicated similar or higher comorbidities in COVID-19 associated ARDS in 2020 compared to non-COVID-19 cases [10,11,12], our study results demonstrate that COVID-19 patients in 2021 had notably less comorbidities compared to non-COVID-19 ARDS patients in 2021 and 2019, except for diabetes. Particularly, COVID-19 patients had significantly lower rates of cardiovascular diseases, such as myocardial infarction, congestive heart failure, liver disease, and cancer. These findings are consistent with several observational cohort studies comparing COVID-19 and non-COVID-19 ARDS [10,11].

In a scientific report from 2022, Ahlström and colleagues analyzed the impact of comorbidities and demographics on the 60-day mortality of ICU patients in Sweden, including COVID-19 patients and ARDS patients, using logistic modeling. In their analysis, they found that most demographics and comorbidities did not have a greater impact on mortality in COVID-19 ARDS than in non-COVID-19 ARDS patients. Exceptions included age, which had a higher association of 60-day mortality in COVID-19 ARDS than in non-COVID-19 ARDS patients, and chronic renal failure, which had a protective effect on mortality in ARDS patients, but no effect on COVID-19 ARDS patients [25]. We performed a similar analysis using logistic regression to compare the relative impact of comorbidities on the in-hospital mortality of COVID-19 ARDS and non-COVID-19 patients (Figure 2). Like Ahlström and colleagues, we found that most comorbidities in our study were not of greater importance in COVID-19 ARDS than in non-COVID-19 ARDS patients. A higher age, cerebrovascular disease, and any malignancy significantly increased the odds of dying in both cases. In contrast to Ahlström, we found that chronic renal failure increased the odds of dying in COVID-19 ARDS patients and had no effect on mortality in non-COVID-19 ARDS patients. This finding may be explained by several studies that have shown patients with chronic kidney disease to have a high risk of developing adverse events and dying due to a COVID-19 infection [31]. Notably, congestive heart failure and dementia also increased the odds of dying only in COVID-19 ARDS patients.

### 4.2. Treatment

The current study reflects the change in the use of non-invasive ventilation (NIV) and nasal high-flow therapy since the beginning of the pandemic. While at the beginning of the pandemic several official guidelines recommended early intubation to prevent infection and the transmission of viruses [32,33,34,35], NIV and high-flow therapy have become more popular as alternatives to mechanic ventilation for patients with mild or moderate acute respiratory distress syndrome over the course of the pandemic. This is because several studies showed that the early use of NIV and NHF therapy instead of intubation in COVID-19 patients with mild and moderate hypoxemia may improve outcomes and reduce the need for invasive mechanical ventilation [36,37,38]. Current guidelines in Germany recommend to try high-flow oxygen therapy (HFNC) or non-invasive ventilation (NIV) in patients with COVID-19 and mild or moderate hypoxemic respiratory insufficiency under continuous monitoring and with constant readiness for intubation [5]. The German guidelines on non-invasive and invasive ventilation give weak recommendations regarding the use of high-flow therapy in mild and moderate ARDS and non-invasive ventilation in patients with mild acute respiratory distress [39,40]. A study by Karagiannidis et al. in 2022 showed that there was a significant increase in the utilization of NIV during the 2020 autumn and winter period of the coronavirus pandemic in Germany [41]. Our study shows that COVID-19 ARDS patients were ventilated significantly more often via non-invasive ventilation or nasal high-flow therapy than patients with non-COVID-19 ARDS in 2021 and 2019. Furthermore, we observed an increase in non-invasive ventilation and nasal high-flow therapy even in the non-COVID-19 group from 2019 to 2021. This indicates that non-invasive ventilation strategies have been used more generally over the last couple of years. The utilization of ECMO treatment showed no significant differences between COVID-19 ARDS and non-COVID-19 ARDS patients in 2021. Thus, ECMO treatment seems to have been similarly allocated to COVID-19 and non-COVID-19 ARDS cases. The higher percentage of ECMO in 2019, however, might indicate that there possibly was a higher need for ECMO in 2021 than could be addressed.

### 4.3. Adverse Events and Outcomes

Complications in patients with ARDS are common and include, among others, shock, barotrauma, nosocomial infections, and multi-organ failure [42]. Acute kidney failure is an important adverse event in COVID-19 ARDS and ARDS patients in general. A previous study on the early onset of kidney failure within 48h of invasive mechanical ventilation reported a significantly lower incidence in COVID-19 ARDS compared to non-COVID-19 ARDS patients [43]. This is consistent with the findings of our study that showed a higher incidence of severe acute kidney failure and dialysis in the non-COVID-19 cohort in 2019 and 2021 in comparison to COVID-19 ARDS patients in 2021 [43]. Besides acute kidney failure, we reported a higher incidence of cardiac, gastrointestinal, and respiratory adverse events in non-COVID-19 cases in 2019 and 2021 compared to COVID-19 ARDS cases in 2021.

To date, studies investigating the mortality of COVID-19 ARDS vs. non-COVID-19 ARDS patients have yielded inconclusive results. While some studies showed a higher mortality in COVID-19 ALI/ARDS patients than non-COVID-19 patients [13], others found no difference in mortality between the two groups [44]. A meta-analysis from early 2020 showed a pooled mortality for COVID-19 ARDS patients in all countries of 39% [45]. For non-COVID-19 ARDS patients, on the other hand, cohort studies from the time before the pandemic showed a mortality of approximately 40% [30,46]. Dmytriw and colleagues performed a systematic review of studies that compared the outcome of COVID-19 ARDS and non-COVID-19 ARDS in 2021. Their results showed no significant difference in the 60-day-mortality of both groups [47].

Our study reports an in-hospital mortality rate of 45.9% for COVID-19 ARDS cases in 2021 and 51.2% for non-COVID-19 ARDS cases in 2021, with a rate of 48.6% in 2019 in Germany. This mortality rate is higher than reported elsewhere, such as in a study that analyzed 105 COVID-19 ARDS cases in 2020 in Germany, which had a mortality rate of 34.9% [15], but is similar to another study that used health claims data to analyze the mortality of COVID-19 ARDS patients in 2020, with a rate of 48.3% [16].

### 4.4. Strengths and Limitations

This study possesses several notable strengths. Firstly, it utilized a large sample size for both the COVID-19 ARDS and non-COVID-19 ARDS groups. Furthermore, the inclusion of non-COVID-19 case validation in two different years strengthened the robustness of the analysis. However, there are also some important limitations to consider. First, we only analyzed a sample of 10 percent of the available health claims data in Germany, which may have introduced sampling bias despite efforts to demonstrate representativeness. Second, health claims data can be subject to inaccuracies and omissions, which could impact the validity of the results. This study relied exclusively on disease (ICD) and procedure (OPS) codes, which are primarily used in Germany for medical billing and quality measurement in healthcare. While these codes provide valuable information and are regularly checked for validity by the health insurance companies, they are not able to catch important measures such as laboratory results, symptoms, intubation criteria, and respiratory settings. These things have to be considered when interpreting this study.

## 5. Conclusions

In summary, this study provides a comprehensive overview of the characteristics, comorbidities, treatments, adverse events, and outcomes of patients with COVID-19 ARDS in 2021 and non-COVID-19 ARDS in 2021 and 2019 in Germany. Our findings demonstrate that patients with COVID-19 ARDS in 2021 had a lower incidence of comorbidities, experienced fewer adverse events, and had shorter LOS than those with COVID-19 ARDS. While the mortality rate was lower in 2021 for COVID-19 ARDS patients, it remained comparable to that of non-COVID-19 ARDS patients in 2019. Non-invasive ventilation and high-flow nasal cannula were more common in the COVID-19 ARDS group, but also increased in the non-COVID-19 group from 2019 to 2021. These results highlight the importance of understanding the differences in epidemiology and outcomes between COVID-19 and non-COVID-19 ARDS, which can inform clinical decision making and future research efforts.

## Figures and Tables

**Table 1:** Representativity of the sample of COVID-19 ARDS cases.

Characteristic	10% Sample of COVID-19 ARDS Cases in 2020 ^2^	Population Data of COVID-19 ARDS Cases in 2020 ^1^
age group/age	65–70 (55–60, 75–80)	69.0 (59.0, 78.0)
female	298 (26.94%)	3379 (29.1%)
adipositas/obesity	142 (12.79%)	1464 (12.6%)
infectious pneumonia or abscess of thorax	1071 (96.49%)	11,214 (96.7%)
mechanical ventilation	446 (40.18%)	4747 (40.9%)
ECMO	113 (10.18%)	1307 (11.3%)
LOS	16 (8, 27)	16 (8, 29)
mortality	538 (48.47%)	5604 (48.3%)

^1^ Sagoschen et al., 2022 [16]; ^2^ median (IQR); n (%).

**Table 2:** Patient characteristics, comorbidities, and risk factors in COVID-19 and non-COVID-19 ARDS cases. ^1^ Comparing COVID-19 ARDS 2021 and Non-COVID-19 ARDS 2021, ^2^ Comparing COVID-19 ARDS 2021 and Non-COVID-19 ARDS 2019.

Parameters	COVID-19 ARDS 2021 (*N* = 2654)	Non-COVID-19 ARDS 2021 (*N* = 1274)	Non-COVID-19 ARDS 2019 (*N* = 1486)	*p*-Value (1) ^1^	*p*-Value (2) ^2^
age group	60–65 (55–75)	60–65 (50–75)	60–65 (50–75)	0.7	0.3
female	819 (31.0%)	435 (34.2%)	484 (32.8%)	0.046	0.2
adipositas	462 (17.4%)	197 (15.5%)	183 (12.3%)	0.13	<0.001
emergency	1922 (72.4%)	826 (64.8%)	869 (58.5%)	<0.001	<0.001
Comorbidities					
Myocardial infarction (mi)	144 (5.4%)	98 (7.7%)	150 (10.1%)	0.006	<0.001
Congestive heart failure (chf)	583 (22.0%)	441 (34.6%)	573 (38.6%)	<0.001	<0.001
Peripheral vascular disease (pvd)	160 (6.0%)	134 (10.5%)	169 (11.4%)	<0.001	<0.001
Cerebrovascular disease (cevd)	169 (6.4%)	122 (9.6%)	171 (11.5%)	<0.001	<0.001
Dementia	47 (1.8%)	25 (2.0%)	33 (2.2%)	0.7	0.3
Chronic pulmonary disease (cpd)	400 (15.1%)	218 (17.1%)	267 (18.0%)	0.10	0.015
Rheumatic disease (rheumd)	49 (1.9%)	32 (2.5%)	28 (1.9%)	0.2	>0.9
Peptic ulcer disease (pud)	32 (1.2%)	20 (1.6%)	44 (3.0%)	0.3	<0.001
Liver disease, mild (mld)	108 (4.1%)	99 (7.8%)	128 (8.6%)	<0.001	<0.001
Diabetes w/o chronic compl. (diab)	738 (27.8%)	291 (22.8%)	276 (18.6%)	<0.001	<0.001
Diabetes w chronic compl. (diabwc)	141 (5.3%)	72 (5.7%)	95 (6.4%)	0.7	0.2
Hemiplegia/paraplegia (hp)	112 (4.2%)	83 (6.5%)	124 (8.3%)	0.002	<0.001
Renal disease (rend)	452 (17.0%)	219 (17.2%)	357 (24.0%)	>0.9	<0.001
Any malignancy (canc)	102 (3.8%)	95 (7.5%)	138 (9.3%)	<0.001	<0.001
Liver disease, moderate/severe (msld)	18 (0.7%)	39 (3.1%)	46 (3.1%)	<0.001	<0.001
Metastatic solid tumor (metacanc)	17 (0.6%)	52 (4.1%)	84 (5.7%)	<0.001	<0.001
AIDS	1 (0.04%)	3 (0.2%)	7 (0.5%)	0.10	0.004
Charlson Score	0.0 (0.0, 2.0)	2.0 (0.0, 3.0)	2.0 (0.0, 3.0)	<0.001	<0.001
Risk Factors					
Pneumonia	2639 (99.43%)	1028 (80.7%)	1125 (75.7%)	<0.001	<0.001
Sepsis	1195 (45.0%)	753 (59.1%)	859 (57.8%)	<0.001	<0.001
Trauma	160 (6.0%)	182 (14.3%)	212 (14.3%)	<0.001	<0.001
Aspiration	39 (1.5%)	195 (15.3%)	190 (12.8%)	<0.001	<0.001
Cancer	118 (4.5%)	172 (13.5%)	254 (17.1%)	<0.001	<0.001
Thoracic surgery	37 (1.4%)	78 (6.1%)	115 (7.7%)	<0.001	<0.001
Acute pancreatitis	26 (1.0%)	50 (3.9%)	73 (4.9%)	<0.001	<0.001

**Table 3:** Treatments, adverse events, and outcomes in COVID-19 and non-COVID-19 ARDS cases. ^1^ Comparing COVID-19 ARDS 2021 and Non-COVID-19 ARDS 2021, ^2^ Comparing COVID-19 ARDS 2021 and Non-COVID-19 ARDS 2019.

Parameters	COVID-19 ARDS 2021 (*N* = 2654)	Non-COVID-19 ARDS 2021 (*N* = 1274)	Non-COVID-19 ARDS 2019 (*N* = 1486)	*p*-Value (1) ^1^	*p*-Value (2) ^2^
Treatments					
NIV at all (OPS 8-706)	1567 (59.0%)	529 (41.5%)	523 (35.2%)	<0.001	<0.001
NHF at all (OPS 8-713)	892 (33.6%)	279 (21.9%)	211 (14.2%)	<0.001	<0.001
Invasive (tube/tracheostomy) at all	1865 (70.2%)	943 (74.0%)	1185 (79.7%)	0.015	<0.001
NIV maximum	220 (8.3%)	86 (6.8%)	64 (4.3%)	0.092	<0.001
NHF maximum	291 (11.0%)	68 (5.3%)	31 (2.1%)	<0.001	<0.001
Tube maximum (8-701, 8-704)	1087 (41.0%)	550 (43.2%)	744 (50.1%)	0.2	<0.001
Tracheostomy maximum (5-311, 5-312)	776 (29.2%)	391 (30.7%)	439 (29.5%)	0.4	0.8
Ventilation started prior	209 (7.9%)	134 (10.5%)	142 (9.6%)	0.006	0.063
ECMO treatment (8-852.0/.3/.5/.6)	371 (14.0%)	168 (13.2%)	245 (16.5%)	0.5	0.03
Dialysis (8-853, 8-854, 8-855)	619 (23.3%)	413 (32.4%)	546 (36.7%)	<0.001	<0.001
Blood transfusion (8-800)	949 (35.8%)	704 (55.3%)	890 (59.9%)	<0.001	<0.001
Cardioversion (8-640.0)	171 (6.4%)	124 (9.7%)	148 (10.0%)	<0.001	<0.001
Defibrillation (8-640.1)	23 (0.9%)	27 (2.1%)	46 (3.1%)	0.001	<0.001
Reanimation (8-771, 8-779)	247 (9.3%)	190 (14.9%)	260 (17.5%)	<0.001	<0.001
Adverse Events and Outcomes					
Septic shock	523 (19.7%)	409 (32.1%)	419 (28.2%)	<0.001	<0.001
Cardiogenic shock	62 (2.3%)	117 (9.2%)	152 (10.2%)	<0.001	<0.001
Hypovolemic shock	87 (3.3%)	92 (7.2%)	126 (8.5%)	<0.001	<0.001
Acute kidney failure (Stad 2)	221 (8.3%)	122 (9.6%)	142 (9.6%)	0.2	0.2
Acute kidney failure (Stad 3)	696 (26.2%)	447 (35.1%)	561 (37.8%)	<0.001	<0.001
Myocardial infarction	62 (2.3%)	39 (3.1%)	78 (5.3%)	0.2	<0.001
Cardiac arrest	216 (8.1%)	214 (16.8%)	277 (18.6%)	<0.001	<0.001
Acute liver failure	185 (7.0%)	124 (9.7%)	190 (12.8%)	0.003	<0.001
Acute ulcer/gastrointest. bleeding	82 (3.1%)	58 (4.6%)	90 (6.1%)	0.021	<0.001
DIC	118 (4.5%)	47 (3.7%)	58 (3.9%)	0.3	0.4
Pulmonary embolism	205 (7.7%)	78 (6.1%)	58 (3.9%)	0.069	<0.001
Pneumothorax and lung collapse	349 (13.2%)	315 (24.7%)	342 (23.0%)	<0.001	<0.001
Pleural effusion	191 (7.2%)	181 (14.2%)	260 (17.5%)	<0.001	<0.001
Stroke/Cerebral hemorrhage	82 (3.1%)	51 (4.0%)	85 (5.7%)	0.14	<0.001
Delirium	528 (19.0%)	278 (21.8%)	328 (22.1%)	0.2	0.10
ARDS severity				<0.001	<0.001
Mild ARDS	56 (2.1%)	70 (5.5%)	115 (7.7%)		
Moderate ARDS	517 (19.5%)	247 (19.4%)	310 (20.9%)		
Severe ARDS	2019 (76.1%)	886 (69.5%)	918 (61.8%)		
Indeterminate ARDS	62 (2.3%)	71 (5.6%)	143 (9.6%)		
LOS	17 (10, 29)	19 (9, 34)	21 (10, 37)	0.027	<0.001
LOS ICU	12 (6, 23)	13 (6, 26)	13 (4, 26)	0.5	0.2
Mortality	1219 (45.9%)	652 (51.2%)	722 (48.6%)	0.002	0.10
Transfer to other hospital	678 (25.6%)	231 (18.1%)	327 (22.0%)	<0.001	0.011

## Data Availability

Our research team at BinDoc strives to follow high standards of transparency and reproducibility. That said, the data and script can be made available to reviewers, but the data cannot be made publicly available.

## Supplementary Materials

The following supporting information can be downloaded at: https://www.mdpi.com/article/10.3390/v15061324/s1.
