# Supplementary material for: Same but Different? Comparing the Epidemiology, Treatments and Outcomes of COVID-19 and Non-COVID-19 ARDS Cases in Germany Using a Sample of Claims Data from 2021 and 2019
Source: Viruses. 2023 Jun 5;15(6):1324. doi: 10.3390/v15061324 (PMC10301926; doi:10.3390/v15061324)
Supplement: Supplementary file 1 [file viruses-15-01324-s001.zip › viruses-2417124-supplementary.pdf]

viruses

Supplementary Material

Same But Different? Comparing Epidemiology, Treatments, and Outcomes of COVID and non-COVID ARDS Cases in Germany using a Sample of Claims Data from 2021 and 2019

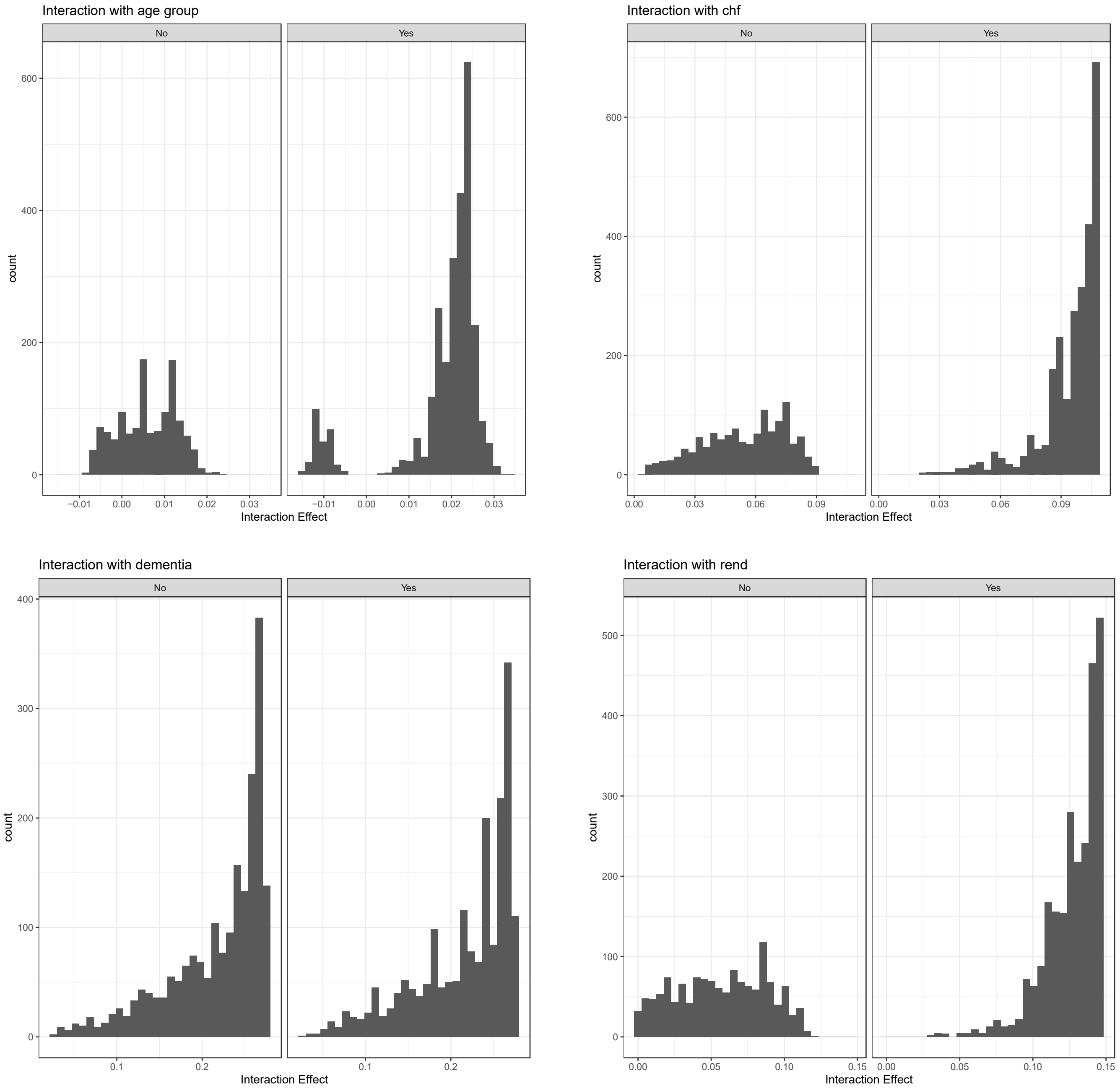

Figure S1. Interaction effects following the logistic regression model in the study (see Figure 1) according to Ai/Norton (2003) and Armstrong (2022), grouped by no significance (No) and significance (Yes) see: https://cran.r-project.org/web/packages/DAMisc/vignettes/binaryDV_interactions.html

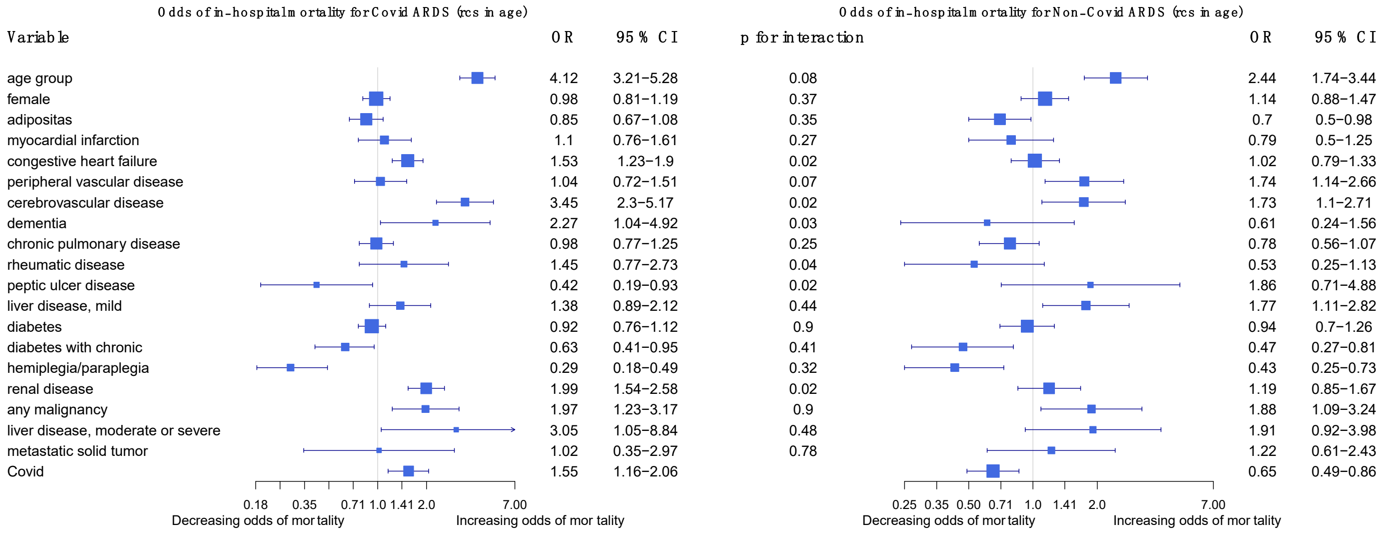

Figure S2. Logistic regression on mortality with comorbidities as predictors, and with restricted cubic splines (rcs) in age variable.

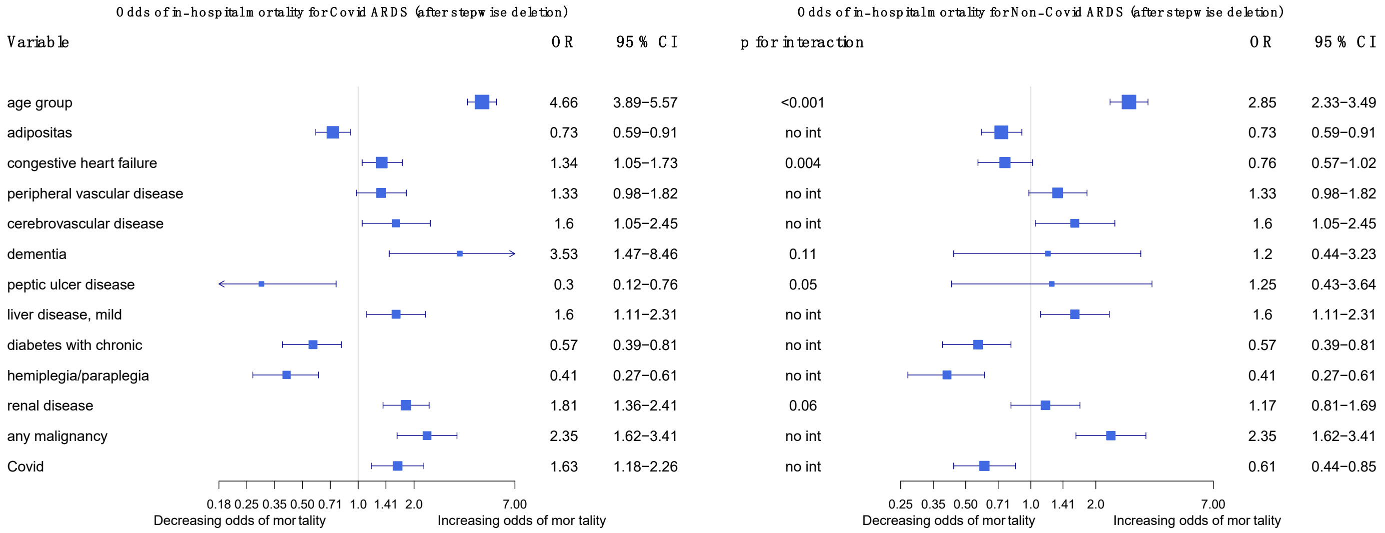

Figure S3. Logistic regression on mortality with prior predictors, plus adverse events, plus treatments (NIV only, NHF maximum, tubus maximum, tracheostomy maximum, and ventilation in other hospital or ambulance), plus severity of ARDS, followed by stepwise forward and backward deletion of covariates. Displayed here: remaining comorbidities after stepwise deletion; no interaction ("no int"), when interaction term was dropped after stepwise deletion.
